# Epithelial Mesenchymal and Endothelial Mesenchymal Transitions in Hepatocellular Carcinoma: A Review

**DOI:** 10.1155/2019/2962580

**Published:** 2019-09-29

**Authors:** Simona Gurzu, Laszlo Kobori, Decebal Fodor, Ioan Jung

**Affiliations:** ^1^Department of Pathology, University of Medicine, Pharmacy, Sciences and Technology, Targu Mures, Romania; ^2^Advanced Medical and Pharmaceutical Research Center (CCAMF), University of Medicine, Pharmacy, Sciences and Technology, Targu Mures, Romania; ^3^Department of Pathology, Clinical County Emergency Hospital, Targu Mures, Romania; ^4^Department of Transplantation and Surgery, Hungarian Academy of Sciences, Semmelweis University, Budapest, Hungary; ^5^Department of Anatomy and Embryology, University of Medicine, Pharmacy, Sciences and Technology, Targu Mures, Romania

## Abstract

**Purpose:**

To present a comprehensive review of the literature data, published between 2000 and 2019 on the PubMed and Web of Science databases, in the field of the tumor microenvironment in hepatocellular carcinoma (HCC). All the data were combined with the personal experiences of the authors.

**Design:**

From 1002 representative papers, we selected 86 representative publications which included data on epithelial-to-mesenchymal transition (EMT), angiogenesis, cancer stem-like cells (CSCs), and molecular background of chemoresistance or resistance to radiotherapy.

**Results:**

Although the central event concerns activation of the Wnt/*β*-catenin pathway, other signal pathways, such as c-Met/HGF/Snail, Notch-1/NF-*κ*B, TGF-*β*/SMAD, and basic fibroblast growth factor-related signaling, play a role in the EMT of HCC cells. This pathway is targeted by specific miRNAs and long noncoding RNAs, as explored in this paper. A central player in the tumor microenvironment proved to be the CSCs which can be marked by CD133, CD44, CD90, EpCAM, and CD105. CSCs can induce resistance to cytotoxic therapy or, alternatively, can be synthesized, de novo, after chemo- or radiotherapy, especially after transarterial chemoembolization- or radiofrequency ablation-induced hypoxia. The circulating tumor cells proved to have epithelial, intermediate, or mesenchymal features; their properties have a critical prognostic role.

**Conclusion:**

The metastatic pathway of HCC seems to be related to the Wnt- or, rather, TGF*β*1-mediated inflammation-angiogenesis-EMT-CSCs crosstalk link. Molecular therapy should target this molecular axis controlling the HCC microenvironment.

## 1. Introduction

Epithelial-mesenchymal transition (EMT) is a process first known to be involved in embryogenesis and tissue repair [1]. In carcinomas, EMT is defined as the transformation of the epithelial cells in cells with a mesenchymal phenotype [1–3]. The EMT of carcinoma cells, also known as epithelial cell plasticity, usually begins with the loss of epithelial cell polarity and the disintegration of the E-cadherin-related cell-cell adhesive [1]. The acquisition of positivity for mesenchymal markers then induces the increased mobility of the tumor cells and a high risk of lymph node or distant metastases.

Although more than 200 papers appear every year in the English-language literature, regarding the EMT of hepatocellular carcinoma (HCC) cells, the exact pathway and interaction of this process with other particular events of the tumor microenvironment, such as angiogenesis, inflammation, and stemness features, are still poorly understood. The main aim of this review is to synthesize the information in the literature regarding the particularities of the HCC microenvironment, taking into account not only the tissue and circulating biomarkers but also the background of peritumor liver parenchyma.

HCC is the fifth most common cancer, the most common malignant primary tumor of the liver, and the third leading cause of cancer-associated mortality worldwide [2, 4–6]. In some Asiatic regions, such as Taiwan, HCC is the leading cause of cancer-related death [7]. In addition to multifocality (intrahepatic metastases), which is a factor of aggressiveness, it has been proven that HCC is one of the tumors with the highest metastatic capacity and that it has a high risk of recurrence. More than 65% of patients showed metastases at autopsy [2]. As very limited and poorly effective therapeutic options exist for HCC [2], the possible predictive role of EMT for the targeted therapy of HCC is also explored in this paper.

## 2. Methodology

For this review, a systematic search of the literature was undertaken to identify papers reporting data on the particularities of the tumor microenvironment in HCC. The review focused on the molecular biomarkers driving HCC plasticity and the possible prognostic and predictive roles of these markers, which were experimentally proven. One of the purposes was to identify which of the markers, which are assumed to act as potential promoters of aggressiveness, proved to be useful for predicting a patient's prognosis, thus indicating the most appropriate therapeutic regimen. The possible role of the tumor microenvironment in inducing resistance to radiotherapy or sorafenib, classic cytotoxic drugs, or other agents used in clinical trials was also taken into account.

To enrich the abovementioned aim and in turn understand the HCC microenvironment, we have selected, from the PubMed and Web of Science databases, representative publications using the MeSH terms and text words “hepatocellular carcinoma,” “epithelial-mesenchymal transition,” “tumor microenvironment,” “stemness,” and “angiogenesis.” Data assessment was conducted independently by all of the authors using predefined terms.

There were 3497 studies published between January 2000 and August 2019, including 12 papers resulting from personal research or from other databases identified via a manual search.

**Figure 1:**
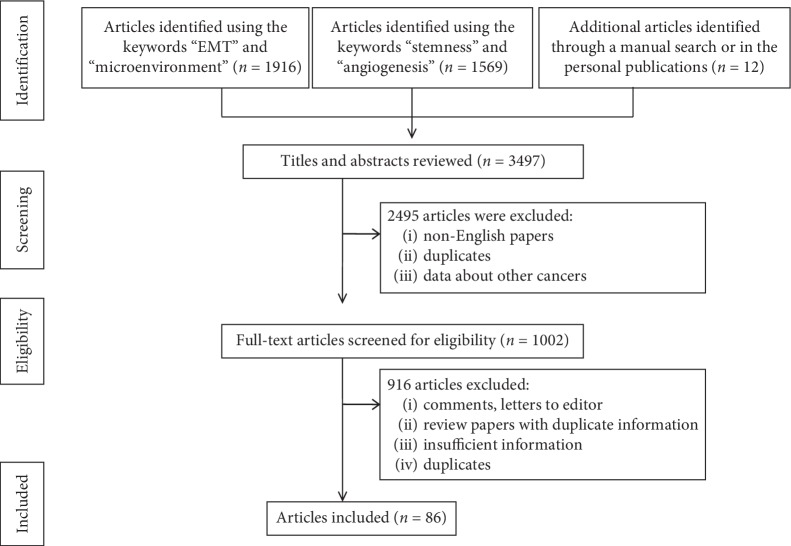
Preferred reported items for systematic reviews and meta-analyses (PRISMA) flow diagram, adapted for data on the tumor microenvironment in hepatocellular carcinoma from the PubMed and Web of Science databases between 2000 and 2019.

After elimination of non-English-language papers, duplicates, or letters, along with noninformative articles (Figure 1), 86 articles were considered to elaborate this review. Besides the clinical studies (*n* = 22), we have also selected those papers in which the clinical findings were further checked by in vivo or in vitro experiments (*n* = 18). At the same time, HCC cell line-based experiments were included (*n* = 21), then, in the same way as the in vitro experiments, in vivo experiments were validated (*n* = 16). As nine review-type articles were considered relevant, they were also selected for in-depth analysis and included in the reference list.

## 3. Molecular Pathways of EMT in HCC

There are several biomarkers that are supposed to be involved in EMT which are independent of the type and localization of carcinomas. The biomarkers expression can be successfully quantified in the tumor cells using immunohistochemical (IHC) methods [1].

EMT is IHC and characterized by a decrease or absence of the transmembrane adhesive of glycoprotein E-cadherin and the E-cadherin-to-N-cadherin (neural cadherin) switch [2, 8–10]. E-cadherin is linked to the actin cytoskeleton via the catenin family (*α*-catenin, *β*-catenin, *γ*-catenin, and p120) [9] and other proteins, such as claudins (types 3, 4, and 7), occludin, ZO-1, desmoplakin, and plakoglobin [1, 2, 8]. EMT is induced by transcription factors that repress the E-cadherin expression. These include Twist1, Twist2, Snail1/Snail, Snail2/Slug, and zinc finger E-box-binding homeobox 1 (ZEB1) and ZEB2 [1, 2, 11–13]. The membrane-to-nuclear translocation of *β*-catenin is also an indicator of EMT, similar to Snail nuclear translocation [7, 8, 13]. The other markers that contribute to the orchestration of EMT include tumor growth factor *β* (TGF-*β*), epidermal growth factor (EGF), platelet-derived growth factor (PDGF), fibronectin, vimentin, hepatocyte growth factor (HGF), tumor necrosis factor (TNF), and ubiquitin regulator A20 [1, 2, 10–13].

As in other carcinomas, the EMT of HCC cells can be regulated by microRNAs (miRNAs) and long noncoding RNAs (lncRNAs) [3, 8, 14, 15]. The miRNAs are small noncoding RNAs comprising 18–22 nucleotides lengthways, which trigger specific proteins and can act as tumor suppressors or oncogenes [1, 14–16]. The lncRNAs are noncoding RNA transcripts that are longer than 200 nucleotides [4, 14].

### 3.1. Wnt/*β*-Catenin Pathway

Similar to other carcinomas, the EMT of the HCC cells appears to be driven by the Wnt/*β*-catenin signaling pathway. In patients with hepatitis-induced HCC, *β-*catenin mutations were reported to occur in 13–41% of cases [7]. In more than 55% of the cases, the mutations occur at the serine/threonine residues in the GSK-3*β* region of the *β-*catenin gene [7]. Codons 32, 33, 34, 41, and 45 of the gene can also be mutational spots [7].

The IHC studies that have taken into consideration this molecular pathway showed a loss of the membrane expression of the adhesive molecule E-cadherin in 17–69% of HCC cases [2, 9, 11, 13]. The membrane expression of *α*-, *β*-, and *γ*-catenin and p120 is also reduced in 76%, 63%, 71%, and 73%, respectively, of HCC cases [9].

The reduced positivity of E-cadherin or other catenins is considered to be an independent indicator of poor survival [9]. Most of the authors admit that E-cadherin expression does not depend on clinicopathological parameters, such as a patient's age and gender, the tumor diameter, the serum level of alfa-fetoprotein (AFP), and the background development of chronic hepatitis or cirrhosis [13]. In other papers, it was proven that reduced positivity of the E-cadherin/catenin complex was inversely correlated with the histological grade of the tumor and directly correlated with the presence of intrahepatic metastasis and capsular invasion, without correlation with satellite nodules [9]. The membrane expression of *α*-, *β*-, and *γ*-catenin and p120 was correlated with tumor size and stage [9]. Of the four catenins, only p120 was found to be correlated with the AFP serum level [9].

Catenins are especially expressed in the cell membrane or cytoplasm but can also enter the nucleus [9]. Nuclear *β*-catenin immunoexpression was reported with large variations, with between 5% and 50% of the cases being found to be positive [5, 11, 13]. Most of the HCC cases showing a diffuse membrane expression of E-cadherin also present membrane positivity for *β*-catenin, but the loss of E-cadherin is usually associated with *β*-catenin nuclear expression [13].

The IHC membrane-to-nuclear translocation of *β*-catenin is considered to reflect the presence of mutations in the CTNNB1 gene, which is an indicator of EMT [7, 13]. *β*-Catenin can be translocated from membrane to nucleus by the adhesion of the Tcf-Lef family of DNA-binding proteins [7]. About 80% of the *β*-catenin mutated cases presented IHC nuclear expression but not all of the cases with nuclear positivity showed *β-*catenin mutations in exon 3 (GSK-3b phosphorylation sites) using the primer sense 5′-AGCTGATTTGATGGAGTTGG-3′ and antisense 5′-ACCAGCTACTTGTTCTTGAG-3′ [7]. Although *β*-catenin nonmembranous expression is considered to be a negative prognostic factor of HCC, this is probably the reason why *β*-catenin mutation has proven, in a few studies, to be an indicator of a favorable prognostic factor related to low-stage (I, II), low-grade, hepatitis B virus-negative (HBV-negative) HCCs that predominately occur in elderly patients with low serum levels of AFP [7]. The rate of *β*-catenin mutations does not depend on the tumor size, uni- or multifocality, or even the presence or absence of cirrhosis [7]. It was suggested that there are two genetically distinguished groups of HCC: mutant nuclear *β*-catenin, with a survival rate of more than five years (over 60%), and wild-type nuclear *β*-catenin HCCs, with a more unfavorable prognosis (a five-year overall survival below 35%) [7, 13]. This hypothesis should be tested among large cohorts.

Snail, Twist, and Slug positivity was reported in 57%, 43%, and 51%, respectively, of primary HCCs [7, 11, 13]. E-cadherin expression was shown to be inversely correlated with Snail and Twist [2, 11] (but not Slug), which are assumed to be the main mediators in the EMT of HCC cells [11]. Although independently regulated, Snail and Twist have experimentally been shown to have the potential for added aggressiveness, independent of Slug expression.

The E-cadherin negativity/Snail/Twist positivity/*β*-catenin nuclear expression could be considered to be an independent negative prognostic factor of HCC and an indicator of a high metastatic capacity [2, 11].

N-cadherin marks about 17% of HCC cases and shows membrane IHC expression with/without associated cytoplasmic positivity [13]. The correlation between E-cadherin and N-cadherin is rejected by most of the authors [13], proving that N-cadherin is not a key player for the EMT of HCC cells.

It was recently demonstrated that Wnt signaling can be activated by noncatenin proteins such as MUC13 [17] and collagen triple helix repeat containing 1 (CTHRC1) [18]. MUC13 can be detected in over 40% of HCCs and is correlated with tumor size and stage, encapsulation, venous invasion, and poor outcome [17]. MUC13 seems to induce *β*-catenin phosphorylation at Ser552 and Ser675 sites and, subsequently, *β*-catenin nuclear translocation [17].

CTHRC1 inhibits collagen 1 and stimulates the migration of HCC cells and EMT via PI3K/Akt/ERK/CREB/Snail/TGF*β*/MMPs (matrix metalloproteinases 2 and 9) signaling [18]. CTHRC1 mRNA is positively correlated with tumor size and stage, microvascular invasion, and intrahepatic metastasis [18].

Vimentin positivity is an independent indicator of EMT, early recurrence, and risk of lung metastases and a poor prognosis of HCC [19].

### 3.2. c-Met/HGF/Snail Pathway

HGF is encoded by the MET proto-oncogene [3, 8]. Its receptors stimulate the EMT markers, such as c-MET [19] and growth factor receptor tyrosine kinase (RTK) [2, 11], via the c-MET/Snail pathway [3, 8]. The suppressor of cytokine signaling 1 (SOCS1) was recently shown, in HCC lines, to regulate the HGF signal; SOCS1 inhibited the HGF-induced MET-mediated cell growth/proliferation, the invasion of the extracellular matrix, and the dissemination of tumor cells [2]. The HGF/MET axis also interacts with other biomarkers, such as integrins, semaphorins, EGFR, HER2, or the proapoptotic receptor, FAS [12].

Only a few complex studies have taken into account the IHC expression of c-MET in HCC [20, 21]. They revealed that the c-MET overexpression should be considered to be an independent negative prognostic factor, indicating early recurrence and poor survival [20]. The c-MET overexpression appears to be more frequent in poorly differentiated HCCs and correlates with *β*-catenin nuclear expression [21]. These aspects reveal an interaction between Wnt/*β*-catenin and c-Met/HGF/Snail pathways. There is no consensus regarding the best method and system for the IHC quantification of c-MET expression [20].

### 3.3. Notch-1/NF-*κ*B Pathway

NF-*κ*B is a transcription factor that can be activated during the EMT of several carcinomas, including HCC [22, 23]. It exerts an antiapoptotic effect via the Notch-1/NF-*κ*B pathway and interacts with the genes involved in apoptosis, such as Bcl-2, cyclin D1, survivin, and cIAPs (cellular inhibitor of apoptosis) [22, 23]. The NF-*κ*B is suppressed by TNF which is encoded with the TNFAIP3 gene [23]. NF-*κ*B is also known as the ubiquitin regulator A20 or alpha-induced protein 3 [23].

### 3.4. TGF-*β*/SMAD Signaling

In HCC, TGF-*β* can act as an autocrine or paracrine growth factor or can exert an extrinsic activity which induces a change in the tumor microenvironment [24]. TGF-*β* interacts with the extracellular matrix metalloproteinase, MMP3, and appears to be downregulated by agents such as CR6-interacting factor 1 (CRIF1) [25]. CRIF1 also regulates the genes PTEN, SMAD (2, 3, and 6), and CDK6 and induces EMT via decreased E-cadherin and the upregulation of Twist, N-cadherin, and Snail [10, 24, 25].

### 3.5. Basic Fibroblast Growth Factor- (bFGF-) Related Signaling

In vitro, the complex bFGF and its receptors induced EMT and the metastasis of HCC cells via activation of the AKT/GSK-3*β*/Snail/Twist1 signaling pathway [26].

### 3.6. miRNAs Targeting the EMT-Related Biomarkers

Although miRNAs are described as attractive therapeutic targets, the molecular mechanisms of their signals are still unknown [3, 8].

The Met/Snail signal is suppressed by miR-148a [3]. Its expression is decreased in HCC compared with normal liver parenchyma, with a more significant loss in cases with portal vein tumor thrombosis [3]. In human HCC, miR-148a expression has been shown to be directly correlated with the mRNA level of the E-cadherin gene and inhibits the expression of other EMT markers, such as fibronectin, N-cadherin, vimentin, and nuclear Snail [3].

Similar to miR-148a, miR-449a inhibits EMT via the Met/Snail signal, but other targets (e.g., Bcl-2, cyclin D1, E2F3, Notch1, KLF4, and androgen receptor) can also be involved [2, 8]. Its decreased expression was also more frequently found in cases with portal vein tumor thrombosis, with the overexpression of miR-449a supposed to inhibit cell motility, reduce the nuclear accumulation of Snail, and decrease the rate of occurrence of pulmonary metastases [8].

miR-1271 targets the forkhead box Q1 (FOXQ1) protein, which appears to be involved in EMT. This miRNA was recently proven to be downregulated in HCC, compared with normal liver parenchyma [14]. In other carcinomas, FOXQ1 was demonstrated to be a target of TGF-*β*- (e.g., breast cancer) or the Wnt-*β*-catenin signaling pathway (e.g., colorectal cancer) [1, 14]. Although miR-1271 induced apoptosis in HCC lines, its role in the genesis and evolution of this hepatic tumor is still unknown [14].

Other supposed HCC-related miRNAs are miR-26a, miR-26b, miR-101, miR-122, miR-124, miR-150, miR-181 (expressed in *α*-fetoprotein-positive HCCS), miR-195, miR-199a, miR-216a/217, and miR-331-3p [6, 10, 27–31]. The downregulation of miR-124 and miR-26b induces the EMT-related aggressive behavior of HCC [10]. miR-124 negatively regulates the oncogenes ROCK2 and EZH2 [10]. miR-150 directly targets ZEB1 and two proteins involved in DNA repair (MMP14 and MMP16) [28, 32]. MMP16 induces E-cadherin loss and directly correlates with the overexpression of the mesenchymal markers, vimentin, and N-cadherin, at both mRNA and protein levels [32]. miR-195 is a member of the miR-15 family [29] which is favored by downregulation in the occurrence of lung metastasis by targeting the FGF2 and vascular endothelial growth factor A (VEGF-A) genes [30]. miR-199a regulates E-cadherin expression via Notch1 direct targeting [31].

In the most recent studies, the signature sets of miRNAs are described as being involved in HCC genesis. In nonalcoholic steatohepatitis-associated HCC cell lines, a panel of 10 miRNAs was experimentally proven to suppress the most frequent carcinogenesis pathways, especially Wnt/*β*-catenin and TGF-*β*, the signal transducer and activator of transcription 3 (STAT3), extracellular signal-regulated kinase 1 (ERK/MAPK), PPAR*α*/RXR*α*, PTEN, RAR, cell cycle regulation, stem cell regulation, c-myc, and the mechanistic target of rapamycin (mTOR) and amphiregulin (AREG), EGF, and NF-*κ*B signaling [15]. These 10 miRNAs were identified as hepatocarcinogenesis suppressors: miR-17-5p, miR-221-3p, miR-93-5p, miR-25-3p, miR-181b-5p, miR-106b-5p, miR-186-5p, miR-222-3p, miR-15b-5p, and miR-223-3p [15].

In HCC developed in patients with cirrhosis, a panel of 12 miRNAs was proposed to influence carcinogenesis and tumor progression [16]. The upregulation of miR-221 and miR-222 in HCC samples, compared with cirrhosis, was a common event [16]. These miRNAs trigger the CDK inhibitors p27 and p57 and the PI3K-PTEN-AKT-mTOR signaling pathway [16]. miR-106b, miR-21, miR-210, miR-224, miR-34a (target of p53), miR-425, miR-519a, miR-93, and miR-96 were also upregulated, whereas let-7c was downregulated in HCC, compared with normal or cirrhotic liver parenchyma [16].

The five miRNAs, which were common to the two studies involving HCC lines derived from nonalcoholic steatohepatitis and cirrhosis, were miR-34a, miR-93, miR-106b, miR-221, and miR-222 [15, 16]. Independent of the previous aspect of liver parenchyma, miR-21, miR-221, miR-222, and miR-224 appear to be predominately overexpressed in HCC and can serve as therapeutic targets [15, 16]. On the other hand, miR-210, miR-220, miR-224, miR-425, and miR-519a were hypothesized to be more HCC-specific [16]. In the most recent studies, the miRNA signature was hypothesized to influence the speed of HCC cells proliferation. In fast-growing HCC, downregulation of E-cadherin was associated with EMT via upregulation of five miRNAs, namely, miR-15b-5p, miR-421, miR-1303, miR-221-3p, and miR-486-5p [33].

### 3.7. lncRNAs and the EMT of HCC Cells

lncRNAs have been shown to influence the progression of HCC and to promote invasive capacity [28, 34–36], especially in HBV-related tumors [4, 37], but the understanding of their role in EMT is still incomplete. Several lnRNAs are described as being involved in HCC progression: HOTTIP, HOXA13, MALAT1 (metastasis-associated lung adenocarcinoma transcript 1), HOTAIR (HOX transcript antisense RNA), HULC (highly upregulated in liver cancer), MEG3 (also known as GTL2), ZFAS1, ZEB1-AS1, ZEB2-AS1, Linc00974, Linc00261, H19, DANCR, TCF7, Dreh, MVIH, HEIH, LET, ATB, ITGB1, antisense Igf2r (AIR), CCAL, uc002mb, and PVT-1 [28, 34–41].

In HCC tissue, CCAL overexpression is associated with a larger tumor size, an advanced pTNM stage and a low apoptotic rate; it induces EMT via the Wnt/*β*-catenin pathway activation [40]. HOTAIR is also overexpressed compared with normal parenchyma and induces aggressiveness in tumor cells [41]. HOTAIR inhibits the mismatch repair (MMR) proteins, MSH2 and MSH6 and, as result, enhances the microsatellite instability (MSI) status of HCC cells [41].

Linc00261 is decreased in HCC tissue compared with normal liver parenchyma [39]. Its decreased level might induce EMT via activation of the Notch-1/NF-*κ*B pathway and is correlated with tumor size, TNM stage, and low survival rate [39].

The first lncRNA described as influencing the EMT of HBV-induced HCC was HULC; a single nucleotide polymorphism, such as rs7763881, may induce EMT [40, 42, 43]. ZEB2-AS1 upregulation induces metastatic ability via the downregulation of E-cadherin and the upregulation of vimentin [37]. Recently, it was experimentally demonstrated that the HCC core of lncRNAs includes the following five lncRNAs: FABP5P3, LOC100996735, LOC100996732, ZEB1-AS1, and ZFAS1 [28]. The most upregulated lncRNA was found to be ZFAS1 [28]. As ZFAS1 contains a site for miR-150, which targets ZEB1 (which regulates E-cadherin), MMP14, and MMP16, we can suppose that ZFAS1 might play an important role in the EMT of HCC cells via matrix metalloproteinases and the Wnt/*β*-catenin pathway [28].

EMT can also occur via the IL-6/STAT3/lncTCF7 signaling axis [38].

## 4. Cancer Stem-Like Cell Biomarkers

Similar to other carcinomas, the EMT pathways are commonly driven via the activation of cancer stem-like cells (CSCs) [44–46]. These are also known as progenitor cells or tumor-initiating cells (TICs) and present self-renewable capacities [44–48]. The CSCs may activate the Wnt/*β*-catenin pathway and induce chemo/radiotherapy resistance, disease relapse, and metastasis [46, 47] and are also responsible for tumor heterogeneity [48].

The markers that have been proven to act as hepatic CSCs are A6, OV6, CD133 (also known as prominin-1), CD44 standard isoform (CD44s), CD90, CD45, CD13, CD24, cytokeratin 19 (CK19), the epithelial cell adhesive molecule (EpCAM, also known as CD326), octamer-binding transcription factors (Oct3/4), aldehyde dehydrogenase-1 (ALDH1), SOX2, nestin, C-KIT, and CD105 (also known as endoglin) [12, 19, 27, 43, 44, 49–53]. The CSC-related genes are Notch, *β*-catenin, and Oct3/4 [27]. In an experimental study that investigated the mRNA expression of 12 EMT-related/stemness markers (CD133, CD90, CD44, ALDH1, CK19, OCT4, SOX2, vimentin, nestin, CD13, and EpCAM), only CD44 and CD133 proved to be upregulated in HCC cells, compared with normal hepatic parenchyma [49].

The cell surface adhesive, glycoprotein CD44s, is not expressed in the normal mature hepatocytes but marks over 55% of HCC cells [45, 49]. The positivity of CD44 is directly correlated with Twist 1 overexpression [27, 45] and interacts with the HGF/MET or TGF-*β* molecular axes [12, 27, 45]. Although the prognostic value of CD44 is controversial [45], a meta-analysis comprising 14 studies with more than 2200 patients showed that CD44 expression was directly correlated with the pTNM stage but not with the tumor grade or AFP serum level [47]. Its positivity was shown to be an indicator of poor overall survival, but the association with disease-free survival was rejected [47]. The heterogeneity of the reported results is based on the use of several clones/isoforms (CD44, CD44s, and CD44v6) and a lack of consensus regarding the cutoff value (which was reported as at least one positive cell or a value of 10%, 25%, or 50%, respectively) [47].

CD133 has been shown to be a CSC hepatic marker since 2007 [43]. It marks over 25–50% of HCC cells [49, 52]. CD133-positive HCCs are more aggressive, express CSC-related genes, and present low overall survival [27, 44, 49]. Some studies rejected the independent prognostic role of CD133 [52].

CD90 is especially expressed in poorly differentiated HCCs [27]. Of all of the stemness markers, it appears to be the one that is most involved in inducing lung metastases [52] and can coexist with c-KIT, CD105 (endoglin), and FLT1 positivity [48, 53]. About 40% of HCC showed CD105 positivity in the tumor cells as an indicator of microvascular invasion and poor recurrence-free survival [53].

No standard cutoff value is known for CD133, CD90, or other CSCs markers. We consider that 10% should be the cutoff value for the IHC quantification of all CSC markers and the stromal expression should also be taken into account as a prognostic indicator. We also agree with the use of the three scores utilized by Zhao et al.: score 0 (no stained or < 10% stained cells), score 1 (11–50% stained cells), score 2 (51–80% stained cells), and score 3 (>80% stained cells) [49]. Moreover, CD44 variant isoforms (CD44v8-10) should not be used to study HCC behavior [45], while an HCC stem cell should not be defined based on its IHC positivity for only one of the CSC markers [45]. To define a CSC and establish its prognostic value, double positivity for CD44s/CD133 or CD44s/CD90 is required [27, 45, 49].

Double positivity for CD44s and CD90 was proven to be associated with CD45 negativity and a higher aggressiveness, compared to only CD133-positive HCC cells [27]. Double positivity for CD44/CD133 was found in over 36% of HCC cases and demonstrated to be a strong negative prognostic indicator [49]. Double positivity for CD90/CD105 can be an indicator of EMT associated with endothelial-mesenchymal transition (End-MT); this can confirm the vasculogenic mimicry or the possible role of CD105 as a CSC [10, 53].

The CSC marker, CD13, is overexpressed in one-third of HCCs and considered to be a marker of semiquiescent HCC cells [19, 27]. Although its positivity was proven to be a negative prognostic factor, especially in patients with large tumors, no correlation with E-cadherin or vimentin was emphasized [19]. The cell division rate appears to be influenced by the expression of CSC biomarkers. CD13(+)/CD90(−) cells are mainly in the G0/G1 phase, and CD13(+)/CD90(+) cells are in the S-to-G2/M phase, whereas CD13(−)/CD90(+) cells are more frequent in the G2/M-to-S phase [44].

The epithelial cell adhesive molecule, EpCAM (CD326), is considered to mark epithelial CSCs [41]. EpCAM appears to increase the invasiveness potential of tumor cells as well as the risk of portal vein invasion [27]. The CSCs' proliferation rate is influenced by lncRNAs such as HOTAIR [41].

The exact mechanism of the CK19-inducing aggressiveness of HCC and its relationship with CSCs are unclear [51]. In normal liver parenchyma, CK7 and CK19 are not expressed; they mark the bile duct cells [50]. The normal hepatocytes usually express CK8 and CK18 [50]. Some studies have confirmed that about one-third of HCCs are CK7(+)/CK19(−) [50]. CK19 marks 11–31% of HCCs [50, 52], and the coexpression of CK7 and CK19 was described in 9% of HCCs [50]. CK19 and/or CK7 positivity is an indicator of the high risk of recurrence and low overall survival [50–52]. CK19 positivity is directly correlated with tumor size and portal vein invasion [51]. The HCC cells marked by biliary markers might occur as the aberrant differentiation of CSCs [50, 51]. This aspect was experimentally proven by the self-renewal capacity of CK19-positive cells, which were capable of transforming into CK19-negative cells and induced EMT via TGF*β*/SMAD signaling [51]. CK19 can be coexpressed with TGF*β* and EpCAM, especially in large tumors [51].

## 5. Circulating Tumor Cells

In the peripheral blood of patients with HCC, the EpCAM-based identification of circulating tumor cells (CTCs) is considered to be an indicator of portal vein thrombosis, early recurrence risk, and high metastatic potential [33, 45, 54–57].

The mechanism for the survival of CTCs is still unclear. They can be epithelial on release but acquire a mesenchymal or an intermediate phenotype (a hybrid cell that expresses both epithelial and mesenchymal markers; also known as the semimesenchymal cell) during hematogenous transit [54, 55, 57]. A mesenchymal phenotype might protect them from apoptosis, anoikis, and immune mechanisms [54, 55, 57]. Smad-induced Wnt signaling activation was proposed to be involved in the EMT of hepatic CTCs [57].

These CTCs are marked by DAPI and the IHC biomarkers pan-CK, CDH1, and hepatocyte-specific antigen (HSA) and negative for the leukocyte markers CD45 and CD16 [56, 57]. More than 80% of CTCs express vimentin, Twist, Smad, and CTNNB1 as indicators of EMT [56, 57]. The positivity rate for Twist and vimentin is correlated with tumor size and TNM stage but not with the number of tumors [56]. The vimentin-positive CTCs were more frequently detected in patients within Milan criteria, compared with those beyond Milan criteria [56]. Other transcription markers, such as ZEB1, ZEB2, and Snail, can be detected in the CTCs without prognostic value [55]. E-cadherin and Slug did not mark the hepatic CTCs [56].

CD44s-positive HCC circulating cells confirmed EMT during the metastatic step; the mesenchymal phenotype is even more expressed in CD44s(+)/CD90(+) cells [44]. Some of the EpCAM-positive CTCs can be negative for CSC markers such as CD90 [44].

bFGF-related EMT was proven by an increase in serum bFGF in patients with HCC compared with healthy volunteers and a decrease compared with patients with chronic hepatitis and/or cirrhosis [26, 58]. Circulating TGF-*β* level was shown to be increased in patients with fast-growing HCC, compared with slow HCC [33].

Due to the spatial heterogeneity of CTCs, it was suggested that they should be counted in the hepatic vein, where they are in clusters; these cells are more isolated in the peripheral veins [57]. In the hepatic vein, the epithelial and intermediate phenotypes predominated compared with the more frequent mesenchymal cells detected in the peripheral veins [57]. As the EpCAM is downregulated during the EMT of CTCs, a low number of CTCs can be detected in the peripheral bloodstream of patients with HCC; they do not reflect the true number of viable cells in circulation [55]. For this reason, novel biomarkers, such as the major vault protein (MVP) [55] and CTHRC1 [18], are proposed for use as a more proper detection of HCC circulating cells with a mesenchymal or an intermediate phenotype [55]. The number of CTCs is positively correlated with the number of mesenchymal cells detected in the HCC tissue using specific IHC markers; they are not correlated with the amount of epithelial or intermediate HCC tissue cells [54].

## 6. EMT and Inflammation

The interplay between inflammation, hypoxia, and EMT seems to be the critical link that shapes the HCC microenvironment [59]. On the one hand, intratumoral interleukins, such as IL-1*β* and IL-6, are correlated with the number of proinflammatory tumor-associated macrophages [38, 59]. At the same time, IL-1*β* mediates the functional maintenance of M2 monocyte-derived macrophages, which play a proinflammatory role and enhance the proliferation and invasion of HCC cells [60]. On the other hand, transactivation of the complexes IL-6/STAT3/lncTCF7 or IL-6/STAT3/Snail-Smad3/TGF-*β*1 promotes the invasion of HCCs developed in patients with hepatitis [24, 38, 61], especially the nonalcoholic type [15].

In cell lines with hepatitis virus C-related (HCV-related) HCC, Twist positivity, an independent negative prognostic marker, is more frequent than it is in HCC developed in non-hepatitis-related carcinomas [11, 58]. In human samples with HCV-related HCC, EMT was found to be driven by the Wnt-*β* catenin pathway, which is probably modulated by some viral proteins, such as NS5A [13], or occurs as a result of bFGF activation [58]. Although it was hypothesized that mutations in the CTNNB1/*β*-catenin gene, exon 3, occur more frequently in patients with non-HBV-related HCC [7], this aspect was not confirmed in all further studies [12]. However, the mutation spectrum appears to be different: codons 33 and 41 were more frequently mutated in patients with HBV-related HCC, whereas in patients with non-HBV-related HCC, codon 45 was the mutational hotspot of exon 3 of the *β*-catenin gene [7]. The rate of mutations within codons 32 and 34 was not dependent on the viral history of the patient; this was similar in both HCV-related and HBV-related HCCs and should be considered as the mutational hotspot of these carcinomas [7].

The distribution of some stemness markers also appears to be correlated with inflammation. CD90 is more frequently expressed in patients with hepatitis-related, compared with non-hepatitis-related, HCCs, whereas CD133-positive HCCs are more frequently non-hepatitis-related [44]. Other studies showed that the coexpression of CD44 and CD133 is not influenced by HBV but that CD133 is more frequently expressed in HCC developed in patients with cirrhosis [49]. In HBx-infected hepatoma cells, TGF-*β* proved to upregulate CD133 expression and induce cancer stemness and EMT [62].

The CD13-positive CSCs are equally distributed in hepatitis-related and non-hepatitis-related HCC cell lines [44]. CK19 positivity is more frequent in HBV-induced HCCs and a negative prognostic factor [50].

HBV induces the mesenchymal phenotype of HCC cells via the Wnt pathway (E-cadherin loss/upregulated vimentin), which is mediated by lncRNAs such as ZEB2-AS1 [37]. In addition to the Wnt pathway, activated c-Src, STAT3, Akt, and Notch1 were also identified as mediators of EMT induced by HBV [37, 63, 64].

## 7. EMT and Angiogenesis

The HGF/MET axis promotes angiogenesis via interaction with proangiogenic factors such as the vascular endothelial growth factor receptor (VEGFR2) and reverse correlation with thrombospondin-1 [12]. Hypoxia stimulates c-MET overexpression in HCC cells [21].

On the other hand, hypoxia-inducing factor 1*α* (HIF-1*α*) proved to enhance the EMT of HCC cells [54, 64]; its expression correlates with IL-1*β*-related inflammation intensity [44]. Although the hypoxia microenvironment may induce EMT, the hypoxia-related EMT cascade cannot be activated without the simultaneous activation of actin cytoskeleton remodeling via the Wnt/*β*-catenin pathway [65–69]. This remodeling process is expressed more in large HCCs due to tumor size (over 5 cm), while portal invasion remains the most important prognostic indicator of these tumors [51, 66]. Hypoxia-related EMT is also linked with the aberrant hedgehog pathway which plays an important role in maintaining the stem cell capacity of tumor cells [62, 64, 66].

Hypoxia could also promote the EMT of HCC cells via Twist1 upregulation [64]. In cell cultures, 24 h of hypoxia is sufficient for inducing architectural disorders of the cells, along with the upregulation of HIF-1*α* and the downregulation of E-cadherin levels in the tumor cells [67].

VEGFA activation via the downregulation of miR-195 is another supposed mechanism for inducing EMT-related angiogenesis [30]. VEGF positivity can be found in about 70% of HCCs, especially in early stages of HCC developed in cirrhosis [52, 68].

In mouse models, it was demonstrated that proinflammatory IL-1*β* promoted HCC metastasis and induced poor prognosis [59].

In addition to inducing EMT, TGF*β*1 also appears to play a role in the End-MT of intratumor endothelial cells, via CD133 upregulation [10]. The endothelial marker, CD105, is a coreceptor of TGF*β*1 and has stemness properties, being coexpressed with CD90 but not with EpCAM [53]. HIF-1*α*-related hypoxia is also involved in the maintenance of CSCs, via CD90 and CD133, although the IHC expression of VEGF is not correlated with the stemness markers CD133, CK19, and EpCAM [54, 69].

**Figure 2:**
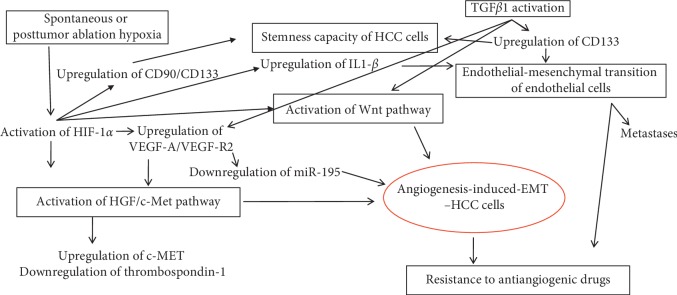
Molecular pathway signaling of angiogenesis-induced epithelial-mesenchymal transition in hepatocellular carcinoma.

Although the metastatic pathway of HCC is not completely understood (Figure 2), it seems to be hypoxia-dependent and is related to the Wnt-mediated or, rather, the TGF*β*1-mediated inflammation-angiogenesis-EMT-CSCs crosstalk link [10, 59].

## 8. Tumor Microenvironment and Therapy

### 8.1. EMT and Chemotherapics

Reducing mortality in HCC strongly depends on the identification of molecular targets that might be used for individualized therapy [2]. The targeting drugs include selective or multikinase inhibitors, as well as antibodies targeting HGF or MET (e.g., DN-30) [11, 12].

Sorafenib, the multikinase inhibitor and antiangiogenic, is currently the only molecular-targeted drug approved by the US Food and Drug Administration to be used as first-line therapy for patients diagnosed with advanced stages of HCCs [10, 12, 69, 70]. Although sorafenib targets the Raf/MEK/ERK signaling pathway and several genes such c-KIT, c-RAF, b-RAF, VEGF-R, c-KIT, and PDGFR*β*, the response rate is low and secondary chemoresistance is frequent [10, 12, 23, 70]. Chemoresistance to sorafenib might be related to the CSCs biomarkers; it is more frequent in those HCCs that express positivity for more than one CSC marker [27, 43, 67, 71]. The CSCs have a quiescent status and can survive after chemotherapy [49]. Experimentally, the CD44(+)/CD133(+) HCC cells proved to be more resistant than CD44(−)/CD133(+) cells [27, 72]. The resistance of CD44(+)/CD133(+) HCC cells might occur as a result of the upregulation of the ATP-binding cassette (ABC) superfamily transporters [73]. Sorafenib proved to decrease the number of CD90(+) cells via c-KIT or TGF-*β* inhibition [48]. As sorafenib upregulates EpCAM expression, PARP inhibitors might be added to target EpCAM + CSCs [48]. For HCCs expressing CD105 in the tumor cells, sorafenib might be combined with the anti-CD105 agent TRC105 (galunisertib), which is currently being tested in a phase II clinical trial [53, 74].

Resistance to cisplatin can be induced by the ABC subfamily member, ABCB1, which forms a complex with STAT3, and also by overactivation of the HOTAIR lncRNA [73]. As HOTAIR enhances the MSI status of HCC cells [41], patients with overexpressed HOTAIR may also be resistant to 5-fluorouracil (5-FU) [74, 75] but may benefit from immunotherapy.

Resistance to classic cytotoxic agents, such as 5-FU and/or adryamicin/doxorubicin/epirubicin, might also be induced by the CSC markers CD13, CD133, CD90, EpCAM, and CK19 [20, 27, 44, 48, 51]. On the other hand, CD13, CD90, EpCAM, CK19, and CD105 might be generated, de novo, after chemotherapy [44, 53]. 5-FU induces EMT via the activation of Snail1 and Snail2 [53].

The anti-VEGFR2 apatinib is an oral drug tested in clinical trials among sorafenib-resistant patients [70]. The oral selective c-Met receptor tyrosine kinase inhibitor, known as tivantinib, is currently being tested as a second-line therapy in a phase II trial, involving patients with advanced HCC and compensated liver cirrhosis [10, 20, 76]. Due to reverse MET-VEGF interaction, it is supposed that antiangiogenic drugs might enhance MET activity [10, 12]. In mouse models, drugs, such as the oral multikinase inhibitor foretinib (with the dual inhibition of angiogenesis and c-MET signaling), proved to successfully deactivate the VEGFR2/MET signaling pathways and induce tumor cells' apoptosis [77].

As the E-cadherin/catenins complex has been shown to be involved in HCC progression, it was suggested that Wnt/*β*-catenin signaling inhibition should be used as a target complex for the synthesis of anti-HCC drugs [9, 78]. The antifibrotic molecule pirfenidone, which is used in patients with idiopathic pulmonary fibrosis, has been experimentally proven to inhibit the proliferation of HCC and to promote apoptosis via *β*-catenin suppression [78].

Inhibition of the other signaling pathways, such as Notch-1/NF-*κ*B, was also proposed for use in EMT-related targeted therapy [23, 24].

The TGF-*β* inhibitor, known as LY2157299, is currently being tested in phase II clinical trials [24, 51, 79]. In experimental studies, LY2157299 has also been demonstrated, in a dose-dependent manner, to induce the dephosphorylation of FAK, b1-integrin, MEK, ERK, AKT, mTOR, and PTEN but not p-38-MAPK-kinase [24, 79]. This drug might be especially useful for the targeted therapy of patients with HCCs that display CK19 positivity [52].

In a phase II clinical trial, a combination of sorafenib with the TGF-*β* inhibitor galunisertib showed acceptable safety and an increased overall survival of over 14 months [80].

In patients with lung metastases, the anti-VEGF drugs should target miR-195 [30]. As miR-195 targets both VEGF-A and bFGF2 [30], EMT might be suppressed by anti-bFGF drugs, such as the oral anti-hyperglycemic agent metformin [26]. Sorafenib proved to inhibit CD90-positive pulmonary metastatic cells [48].

### 8.2. EMT and Radiotherapy

In patients with HCC, radiotherapy is used for the local control of extrahepatic spread or macrovascular invasion [81]. Resistance to ionizing radiation is a characteristic of HCC cells, although the mechanism of induction is still unknown [23, 78]. The most commonly used techniques are radiofrequency ablation, radioembolization, transarterial chemoembolization (TACE), and cryoablation [23, 26, 52, 69, 82, 83]. The newest techniques are three-dimensional conformal radiotherapy, immunoradiotherapy, and image-guided radiotherapy [23, 78, 83, 84]. A combination of chemo- and radiotherapy is also used in advanced HCCs [81, 83].

More than 27% of patients show residual viable tumor cells after TACE [52]. CD105-positive tumor cells, in particular, survive at the periphery of the tumor parenchyma [53]. The radioresistance of HCC cells may be induced via the NF-*κ*B signaling axis [23]. In resistant cells, the inhibition of the NF-*κ*B pathway via enhancing the A20 protein was proposed as a novel therapeutic strategy [23].

In addition to resistance to radiotherapy, TACE-induced hypoxia was shown to produce stromal alteration and the upregulation of stemness markers with a further increased risk of relapse [52]. The IHC studies have reveal an increased intensity and percentage of HCC-positive cells after TACE, compared with the biopsy specimens, especially for CD133, CK19, and EpCAM [52]. The tumor stroma becomes more fibrotic after TACE [52].

After radiofrequency ablation, it was proven that the hypoxic medium might induce the proliferation of stem-like cells, through HIF-1*α*/VEGF-A signaling [69] (Figure 2). These cells showed chemoresistance capacity and increased proliferative and metastatic potential, especially in patients with residual cells after ablation [69].

After insufficient radiofrequency ablation, sorafenib seems to inhibit the EMT of residual cells, via HIF-1*α*/VEGF-A signaling [69, 84, 85]. For this reason, combined radiochemotherapy is recommended to be used [69, 84, 85].

Immunoradiotherapy was recently validated for local HCC. This can be performed using the CD147-targeted agent known as I131-metuximab (I131-mab or CD147-mab) [86]. Although the molecular mechanism is still unknown, the I131-mab appears to inhibit EMT by suppressing the phosphorylation of VEGFR-2 [86].

## 9. Summary and Future Perspectives

Although, in carcinomas, the tumor microenvironment is defined by the old concept of EMT, this has proven to be more challenging for HCC. This comprehensive review of the literature has revealed that similar to other carcinomas, the Wnt pathway is the central event in the EMT of HCC cells, but it does not define the tumor microenvironment. Rather, it is characterized by the interaction between EMT markers and stemness agents. Understanding the molecular pathway of the EMT-angiogenesis-CSCs crosstalk (Figure 2) is mandatory for a therapy that is properly targeted. The EMT markers that deserve further exploration in HCC are E-cadherin and *β*-catenin, which should be correlated with the epithelial stemness marker EpCAM and the mesenchymal CSCs markers CD44, CD133, CD90, and CD105. The molecular mechanism of CK7 and CK19 positivity should also be identified.

The targeted therapy should aim at decreasing hypoxia-mediated stromal changes, especially for large tumors. TACE and radiofrequency ablation should be avoided in large tumors which express CD133, CK19, or EpCAM. In selected cases, radiotherapy should be combined with chemotherapics. The CD90-positive HCCs with pulmonary metastases should be treated with sorafenib, and patients with CK19-positive HCCs should benefit from TGF-*β* inhibitors. In sorafenib-resistant cases, a detailed immunoprofile of tissue cells and CTCs should be used for proper individualized therapy.
